# Electrochemical Immunoassay of *Escherichia coli* O157:H7 Using Ag@SiO_2_ Nanoparticles as Labels

**DOI:** 10.1155/2014/247034

**Published:** 2014-04-27

**Authors:** Guang-Zhu Chen, Zheng-Zhi Yin, Jv-Feng Lou

**Affiliations:** College of Biological, Chemical Sciences and Engineering, Jiaxing University, Jiaxing 314001, China

## Abstract

Silica coated silver (Ag@SiO_2_) nanoparticles were prepared and characterized by transmission electron microscope (TEM) and UV-vis absorption, and the nanoparticles were used as labels in sandwich-type immunosensor of *Escherichia coli* O157:H7 (*E. coli* O157:H7). The labels involved in immunoreaction were dissolved by mixed acid of hydrofluoric acid and nitric acid, and the released Ag^+^ ions were electrochemical stripping analyzed (via differential pulse voltammetry, DPV) at poly(acrylic acid)/poly(diallyldimethylammonium chloride)/carbon nanotubes (PAA/PDCNT) modified glass carbon electrode (GCE), which obviously enhanced the signal of Ag^+^ stripping. Then, the number of *E. coli* O157:H7 could be indirectly reflected by the signal intensity of labeled Ag^+^. And the results showed that the DPV signals were proportional to the logarithm of the *E. coli* O157:H7 concentration in the range from 20 cfu/mL to 8.0 × 10^3^ cfu/mL with the detection limit of 13 cfu/mL.

## 1. Introduction

Pathogenic bacteria, which are distributed in soil, the intestinal tract of animals, and water contaminated with fecal matter, marine, and estuarine water have profound effects on mammals including various infectious diseases [1, 2].* Escherichia coli* O157:H7 (*E. coli* O157:H7) is one of the most dangerous pathogenic bacteria that is of most concern today since a low number of* E. coli* O157:H7 is sufficient to cause severe illnesses, such as hemorrhagic colitis and hemolytic-uremic syndrome, sometimes life-threatening illness, especially in cases involving children and elderly [3, 4]. In view of the fast replication under normal environmental conditions, early detection of* E. coli* O157:H7 in very low numbers is very crucial not only to prevent the consumption of contaminated foods by consumers, but also to alert a foodborne disease outbreak well in advance [5, 6].

Conventional methods for detecting* E. coli* O157:H7 include culture and colony counting methods, polymerase chain reaction (PCR), surface-enhanced Raman spectroscopy, and microarray hybridization, and so forth [7, 8]. Most of these methods are either time-consuming, with low insensitivity, or expensive or require complicated instruments. Therefore, there is an urgent need for developing new methods for detecting* E. coli* O157:H7. In the past several years, immunosensors present an increasing alternative in the detection of pathogenic bacteria for performing simple, sensitive, fast, selective, and cost-effective measurements [9]. In the future, immunosensors should play a greater role in bacterial analysis for food or clinical samples.

Among various immunosensors, electrochemical immunosensor has been quickly developed for detecting biomolecule and microorganism due to the intrinsic advantages of high sensitivity, fast response, low cost, simple instrumentation, and capability of miniaturization. In this field, the study of various labels has attracted researchers' increasing interesting, because it is known that sensitivity of the immunoassay was dependent mainly on labels detection [10–12]. Silver based labels, one of the representative metal based markers [13], have the unique advantages. Electrodeposited silver can be oxidized at relatively positive potential with a relatively sharp peak comparing with other metals including gold, which is favorable to obviating the interference of reducing species and improving the detection precision and sensitivity [14, 15]. On the other side of the coin, the stability and functionalized ability of silver nanoparticles are needed to be further improved.

In this work, the surfaces of silver nanoparticles were coated by a thin silica layer, which could improve the stability, functional sites, and biocompatibility. And the Ag@SiO_2_ nanoparticles were further used as biolabels for sandwich-type detecting* E. coli* O157:H7. The proposed immunoassay exhibited good precision, stability, and reproducibility, which would provide a potential platform for other bacteria and biomolecule detection.

## 2. Experimental

### 2.1. Apparatus and Chemicals

Trisodium citrate, glutaraldehyde, ethanol, 3-Aminopropyltriethoxysilane (APTES), tetraethoxysilane (TEOS), 2,3,4,5,6-pentafluorophenol (PFP), silver nitrate (AgNO_3_), and N,N-diisopropylethylamine (DIEA) were purchased from Shanghai Chemical Reagent Co. (Shanghai, China). Poly(diallyldimethylammonium chloride) (PDDA, 20%, w/w in water, MW = 200,000–350,000), lyophilized bovine serum albumin (BSA, 99%), Tween-20, poly(acrylic acid) (PAA, 35%, w/w in water, MW = 100,000), and 1-ethyl-3-(3-dimethylaminopropyl) carbodiimide hydrochloride (EDC) were purchased from Sigma-Aldrich.* Escherichia Coli* O157:H7 (*E. coli* O157:H7) and anti-*E. coli* O157:H7 were purchased from Ningbo Yuying Pest Control Co. Ltd. (Ningbo, China). Multiwalled carbon nanotubes (CNTs) were purchased from Nanoport. Co. Ltd. (Shenzhen, China) and were pretreated with sonication in mixed acid (*V*_concentrated  nitric  acid_ : *V*_concentrated  sulphuric  acid_ = 1 : 3) for 6 h before use. PBS (0.1 M) with different pH values was prepared by mixing the stock solutions of NaH_2_PO_4_ and Na_2_HPO_4_ and then adjusting the pH with 0.1 M NaOH and H_3_PO_4_. All of the aqueous solutions were prepared in doubly distilled water. All other reagents were of analytical reagent grade and used without further purification.

Electrochemical assay was performed on a CHI 660B electrochemical analyzer (Chenhua, Shanghai, China) with a conventional three-electrode system comprised of platinum wire as the auxiliary electrode, saturated calomel electrode (SCE) as the reference electrode, and glass carbon electrodes (GCE) as the working electrode. The morphologies of the as-prepared samples were characterized by an S-3000 N (Hitachi, Japan) transmission electron microscope (TEM).

### 2.2. Preparation of Ag@SiO_2_ Nanoparticles and Immunosensor Labels

Silver nanoparticles were prepared as follows [16]. 98 mL water containing 30 mg AgNO_3_ was heated to 140°C within 30 min under vigorous stirring. 4 mL freshly prepared trisodium citrate (34 mM) was dropwise added into the AgNO_3_ solution at 140°C with stirring conditions. After further stirring for 1 h, the solution was concentrated to 5 mL.

The SiO_2_ coated Ag nanoparticles (Ag@SiO_2_) were prepared as follows [17]. The obtained Ag solution (3 mL) was added to a mixture of ethanol (100 mL, 99.9%), NH_3_ *·*H_2_O (2 mL, 28 wt %), and pure H_2_O (3.75 mL). After 10 min of ultrasonication, 40 *μ*L TEOS was added, with further stirring for 10 h at room temperature. The suspension was centrifugally separated and washed by ethanol.

Immunosensor labels of Ag@SiO_2_-conjugated anti-*E. coli* O157:H7 were prepared as follows [18]. 20 mg Ag@SiO_2_ nanoparticles was refluxed for 15 h in 4 mL ethanol with 1.5 mL APTES to produce amino groups on silica surface, followed by centrifugation, washing with ethanol and drying at 50°C. 4 mg amino-functionalized Ag@SiO_2_ was dispersed in 2 mL 2.5% glutaraldehyde solution (in 50 mM pH 7.5 PBS) and shaked for 4 h at room temperature. After centrifugation and washing with PBS, the resulting nanoparticles were redispersed in 2 mL PBS containing 50 µg anti-*E. coli* O157:H7 for conjugate reaction for 10 h at room temperature with shaking. The resulting mixture was centrifuged to remove free anti-*E. coli* O157:H7. The obtained sediment (Ag@SiO_2_-conjugated anti-*E. coli* O157:H7) was dispersed in 2 mL PBS of 1% BSA solution for blocked (5 h) at room temperature and centrifuged to remove excessive BSA. The collected complex was dispersed in 2 mL PBS and stored at 4°C.

### 2.3. Preparation of Functional Electrode Surface

5 mg acid-treated CNTs were dispersed into 10 mL 0.1% PDDA aqueous solution containing 0.5 M NaCl, and the mixture was sonicated for 20 min to obtain a homogeneous suspension. The product was centrifuged and rinsed with water to remove residual PDDA. The collected hybrid was redispersed into 10 mL 0.1% PAA solution containing 0.5 M NaCl, following sonication for 20 min and rinsing with water by centrifugation. The obtained PAA/PDCNT hybrid was dispersed in 5 mL water, and 10 *μ*L solution was dropped on the surface of GCE to dry, and PAA/PDCNT-GCE was fabricated.

Afterwards, PAA/PDCNT-GCE was rinsed with ethanol and incubated with a mixture of EDC (0.2 M), PFP (0.2 M), and DIEA (0.2 M) in ethanol for 40 min at room temperature. Then, the electrodes were rinsed with ethanol and were dried in air, followed by incubating with anti-*E. coli* O157:H7 (0.5 µg/µL in PBS) for 10 h at room temperature. Subsequently, the electrode was incubated in 20 *μ*L 2% BSA and 0.05% Tween-20 for 5 h at room temperature and washed with 0.05% Tween-20 and PBS buffer. Then the sensors were stored at 4°C while not in use.

### 2.4. Detecting Procedures

**Scheme 1:**
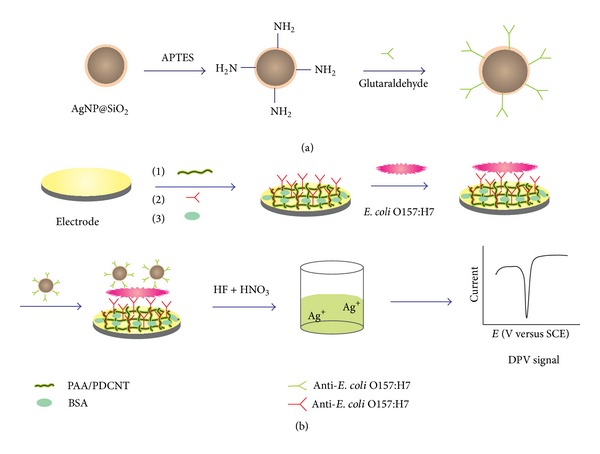
Schematic illustration of the stepwise fabrication process of (a) Ag@SiO_2_-conjugated anti-*E. coli* O157:H7, and (b) the immunosensor.

The analytical protocol is shown in Scheme 1. The immunosensors were firstly incubated with different concentrations solution of* E. coli* O157:H7 for 60 min at 37°C. After the sensor was washed thoroughly with PBS, it was put into the solution of 10 *μ*g/mL Ag@SiO_2_-anti-*E. coli* O157:H7 for 60 min at 37°C. The Ag@SiO_2_-anti-*E. coli* O157:H7 were brought onto the immunosensors' surface by the binding reaction between anti-*E. coli* O157:H7 and* E. coli* O157:H7. After the sensor was washed thoroughly with pH 6.5 PBS to remove nonspecifically bounded conjugates, 15 *μ*L mixture of hydrofluoric acid (0.1 M) and nitric acid (0.1 M) was dispersed onto the electrode and incubated for 10 min to dissolve silver and diluted into 2 mL using PBS. The resulting solution containing released Ag^+^ was electrochemically strripped by differential pulse voltammetry (DPV), which was performed from –0.5 V to +0.5 V with a pretreatment step at –0.5 V for 240 s (DPV :* E*_amplitude_ = 0.05 V, *t*_pulse  width_ = 0.2 s, and *t*_pulse  period_ = 0.5 s).

## 3. Results and Discussion

### 3.1. Characterization

**Figure 1:**
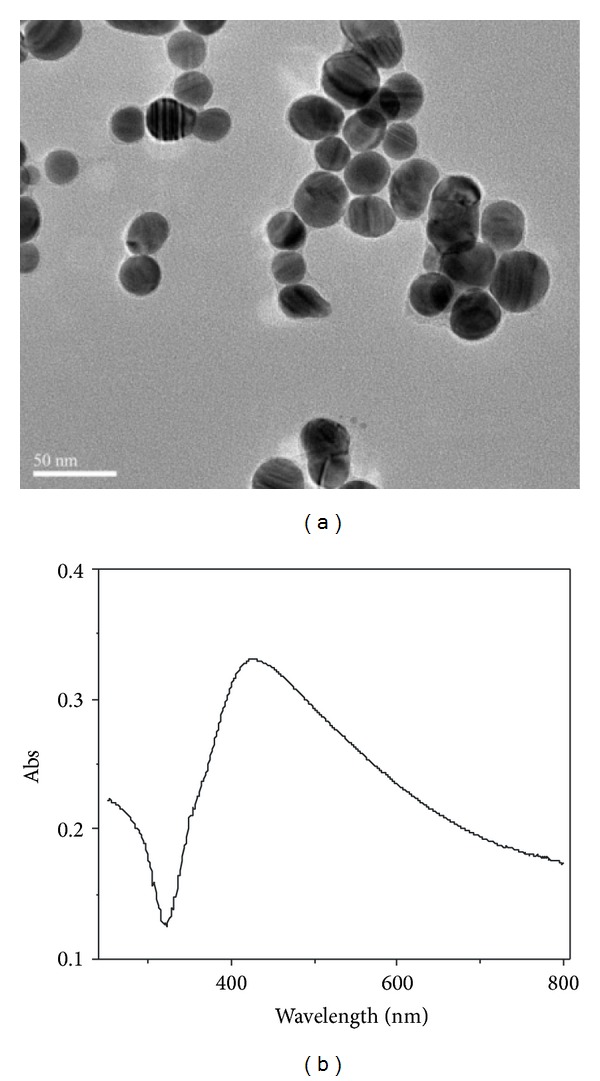
(a) TEM image of Ag@SiO_2_. (b) UV-vis spectrum of Ag@SiO_2_.

The morphology of Ag@SiO_2_ nanoparticles was characterized by transmission electron microscopy (TEM) technique (Figure 1(a)). The TEM images show that the nanoparticles have an average diameter of ∼30 nm, including the very thin silica layers which were coated on Ag nanoparticles.  Figure 1(b) showed the UV-vis absorption spectra of Ag@SiO_2_. It can be seen that the absorption spectra of Ag@SiO_2_ were broad, which may be corresponding to the irregular shape and size of Ag@SiO_2_ nanoparticles.

### 3.2. Optimization of Detection Conditions

**Figure 2:**
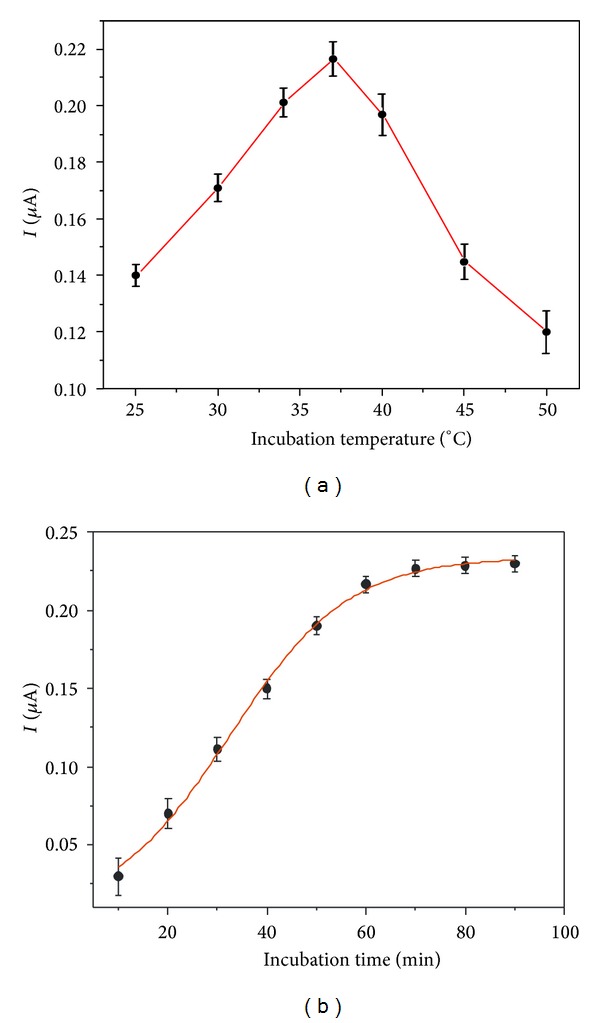
Effects of (a) incubation temperature and (b) incubation time on the stripping current response of Ag^+^ from immunoreaction labels. The concentration of* E. coli* O157:H7 was 500 cfu/mL.

Incubation temperature and incubation time were important factors for the immunological reaction. The effect of incubation temperature was studied in the range of 25 to 50°C via the stripping current of Ag^+^ from immunoreaction labels (Figure 2(a)). It was found that the maximum signal occurred at an incubation temperature of 37°C. When the temperature was higher than 37°C, the peak current reduced, which might be attributed to the decrease of the biomolecule activity at high temperature [19]. Therefore, 37°C was selected as incubation temperature.

According to the study of incubation time influence (Figure 2(b)), the experimental results showed that the peak current increased with increasing incubation time and reached a constant value after 60 min. A longer incubation time did not improve the stripping current response of labeled Ag^+^. Thus, an incubation time of 60 min was chosen for the analysis of* E. coli* O157:H7 using Ag@SiO_2_-labeled antibody.

### 3.3. Dissolution of Labeled Ag@SiO_2_ and Electrochemical Strripping Analysis of Released Ag^+^

The results from the relevance between the electrochemical stripping signal and dissolving time suggested that the labeled Ag@SiO2 could be totally dissolved within 10 min via the mixture of hydrofluoric acid (0.1 M) and nitric acid (0.1 M). For enhancing the stripping signal of dissolved Ag^+^, a systematic study on experimental parameters including the working electrode, deposition potential, and deposition time was optimized.

**Figure 3:**
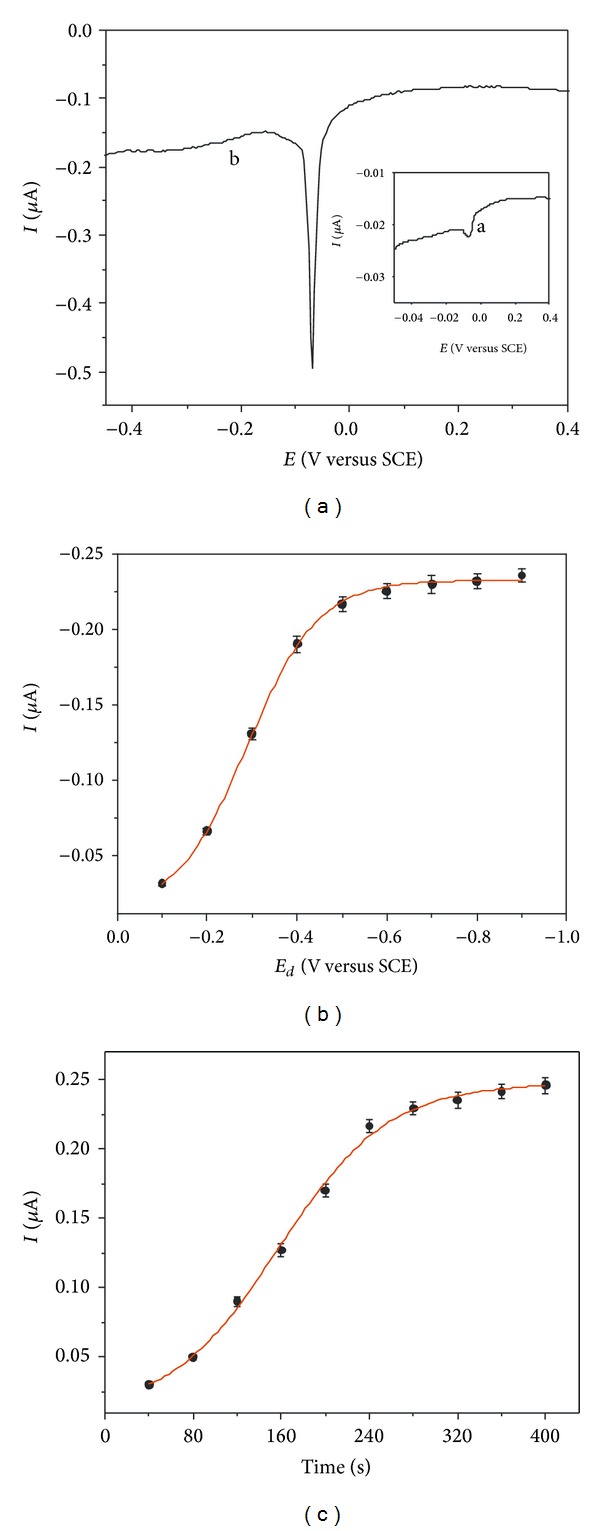
Effects of (a) working electrode, (a) GCE and (b) PAA/PDCNT-GCE, (b) deposition potential (*E*_*d*_), (c) deposition time, on the DPV current of released Ag^+^ from 500 cfu/mL* E. coli* O157:H7 system.

Figure 3(a) shows the stripping current response of released Ag^+^ at bare GCE and PAA/PDCNT-GCE, respectively. It was observed that the stripping current at PAA/PDCNT-GCE was ~100 times higher than the current on bare GCE, suggesting a superior sensitivity by PAA/PDCNT-GCE, which showed that the PAA/PDCNT surface could enrich Ag^+^ efficiently including the formation of Ag^+^-complexes. The relationship between the deposition potential and the stripping current of labeled Ag^+^ was studied from −0.1 to −0.9 V (Figure 3(b)). The results showed that the current intensity increased obviously from −0.1 to −0.5 V, and then more and more little change occurred, nearly a constant value. So, −0.5 V was adopted as deposition potential in stripping process. The influence of deposition time on the stripping intensity of labeled Ag^+^ was depicted in Figure 3(c). As shown, the increasing tendency of peak current was obvious before 240 s, and slight increase happened with prolonging deposition time. Finally, the released Ag^+^ in solution was electrodepositedly preconcentrated via polarizing at −0.5 V for 240 s using PAA/PDCNT-GCE.

### 3.4. Analytical Performance

**Figure 4:**
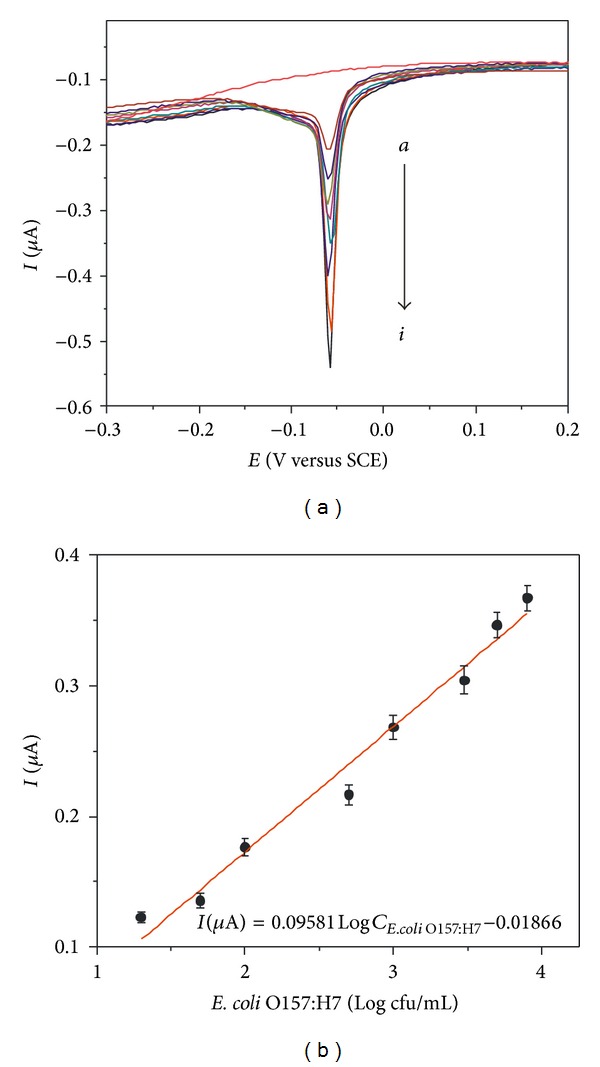
(a) Typical electrochemical DPV curves corresponding to released Ag^+^ from Ag@SiO_2_ labels with increasing concentration of* E. coli* O157:H7 (from *a* to *i*: 0, 20, 50, 100, 500, 1000, 3000, 5000, and 8000 cfu/mL, resp.). (b) Calibration curves of the immunosensor for* E. coli* O157:H7 determination.

Figure 4(a) displays the typical electrochemical stripping response of Ag^+^ from Ag@SiO_2_ labels with different concentrations of* E. coli* O157:H7. And the peak current intensity became higher with the increasing of* E. coli* O157:H7 concentrations. Then, the electrochemical immunosensors were used to detect the concentration of* E. coli* O157:H7. The analytical results showed that a linear relationship between the stripping peak current of labeled Ag^+^ and the logarithmic value of* E. coli* O157:H7 concentration ranging from 20 to 8.0 × 10^3^ cfu/mL was obtained, going along with a slope of 0.09581 and a correlation coefficient of 0.9789 (Figure 4(b)). The further research indicated that the detection limit of the electrochemical immunoassay was 13 cfu/mL for* E. coli* O157:H7 (3*σ*). This value is markedly smaller than 5000 [20], 3270 cfu/mL [2], 1200 [21], 150 cfu/mL [9], 83.7 cfu/mL [22], 30 cfu/mL [4], and 22 cfu/mL [8], and higher than 7 cfu/mL [23], 3 cfu/mL [24], and 2 cfu/mL [25]. The detection limit of 4.5 fg/*μ*L [26] and 800 cells/mL [27] was also realized. The results implied that this detecting approach exhibited a lower detection limit.

The feasibility of applying the sensor in real samples was investigated, and the results were compared with the data from the standard plate count method. Six* E. coli* O157:H7 samples were detected to estimate the precision; ten repetitive measurements were made. The relative deviations of the two methods were from 4.8% to 7.2%. It obviously suggested that there was no significant difference between the results given by two methods. Therefore, the proposed immunoassay could be reasonably applied for* E. coli* O157:H7 determination.

### 3.5. Specificity and Stability of the Immunosensor

The specificity test was conducted by using different microorganisms (*microzyme*,* actinomycetes*). No stripping current response of Ag^+^ was noticed for the above microorganisms at the concentration of 1 × 10^3^ cfu/mL. The results showed that this electrochemical immunoassay could be used to detect* E. coli* O157:H7 in the presence of high concentrations of other microorganisms. And the application of Ag@SiO_2_-anti* E. coli* O157:H7 for* E. coli* O157:H7 detection could play an important role in decreasing the nonspecific adsorption.

The relative standard deviation was 6.1% corresponding to 0.5 × 10^3^ cfu/mL* E. coli* O157:H7 for 8 times determination. When the sensor was stored at 4°C under moist circumstance, the 92.7% signal of its initial response was retained after twenty days. These results further indicated that PAA/PDCNT can provide a biocompatible microenvironment for immobilizing anti-*E. coli* O157:H7 with the bioactivity for the strong interaction with* E. coli* O157:H7. Thus, the developed immunoassay has potential application for* E. coli* O157:H7 determination.

## 4. Conclusion

In summary, we developed a sensitive electrochemical immunoassay for rapid detection of* E. coli* O157:H7 based on Ag@SiO_2_ as anti-*E. coli *O157:H7 labels. The prepared Ag@SiO_2_ expressed fine voltammetric activity, high stability, and good biocompatibility for anti-*E. coli* O157:H7. The Ag@SiO_2_ labels involved in the immunoassay could be sensitively detected by DPV at PAA/PDCNT-GCE after dissolving by the mixture of hydrofluoric acid and nitric acid. A linear relationship between the stripping current response of Ag^+^ and the logarithmic value of* E. coli *O157:H7 concentration was found ranging from 20 to 8.0 × 10^3^ cfu/mL with the detection limit of 13 cfu/mL. These results should be helpful in clinical diagnosis, environmental monitoring, and food security.
